# Quantification of cardiac capillarization in basement-membrane-immunostained myocardial slices using Segment Anything Model

**DOI:** 10.1038/s41598-024-65567-3

**Published:** 2024-07-03

**Authors:** Zhao Zhang, Xiwen Chen, William Richardson, Bruce Z. Gao, Abolfazl Razi, Tong Ye

**Affiliations:** 1 Department of Bioengineering, Clemson University, Clemson, SC USA; 2 School of Computing, Clemson University, Clemson, SC USA; 3 Department of Chemical Engineering, University of Arkansas, Fayetteville, AR USA; 4 Department of Regenerative Medicine and Cell Biology, Medical University of South Carolina, Charleston, SC USA

**Keywords:** Fluorescence imaging, Image processing, Cardiovascular biology

## Abstract

Decreased myocardial capillary density has been reported as an important histopathological feature associated with various heart disorders. Quantitative assessment of cardiac capillarization typically involves double immunostaining of cardiomyocytes (CMs) and capillaries in myocardial slices. In contrast, single immunostaining of basement membrane protein is a straightforward approach to simultaneously label CMs and capillaries, presenting fewer challenges in background staining. However, subsequent image analysis always requires expertise and laborious manual work to identify and segment CMs/capillaries. Here, we developed an image analysis tool, AutoQC, for automatic identification and segmentation of CMs and capillaries in immunofluorescence images of basement membrane. Commonly used capillarization-related measurements can be derived from segmentation results. By leveraging the power of a pre-trained segmentation model (Segment Anything Model, SAM) via prompt engineering, the training of AutoQC required only a small dataset with bounding box annotations instead of pixel-wise annotations. AutoQC outperformed SAM (without prompt engineering) and YOLOv8-Seg, a state-of-the-art instance segmentation model, in both instance segmentation and capillarization assessment. Thus, AutoQC, featuring a weakly supervised algorithm, enables automatic segmentation and high-throughput, high-accuracy capillarization assessment in basement-membrane-immunostained myocardial slices. This approach reduces the training workload and eliminates the need for manual image analysis once AutoQC is trained.

## Introduction

Capillaries in cardiac tissues play an important role in maintaining cardiac function by regulating local blood perfusion and metabolic exchange. Vascular endothelial dysfunction and cardiac capillary rarefaction, have been reported to be correlated with heart disorders, such as diabetic cardiomyopathy^1,2^, dilated cardiomyopathy^3^, hypertrophic cardiomyopathy^4,5^, uremic cardiomyopathy^6^, aging^7–9^, and hypertensive heart disease^10,11^. Evaluation of cardiac capillarization typically involves microscopic examination of cardiomyocytes (CMs) and capillaries in immunostained tissue sections. Quantitative measurements of cardiac capillarization, such as capillary density and capillary-to-cardiomyocyte ratio, are commonly acquired by subsequent microscopy image analysis.

The prevailing method is double immunostaining of CMs and capillaries, followed by thresholding images to segment structures of interest; however, double immunostaining presents challenges during sample preparation, making quantitative image analysis difficult. For example, given limited host species in antibody production (most raised in rabbits and mice), multi-antigen detection, especially in mouse tissues, is problematic due to the high background caused by using antibodies raised in mice. Several protocols and commercial kits have been developed to suppress “mouse-on-mouse” background staining. Their effective applications to specific immunostaining experiments rely on comprehensive optimization and evaluation. Alternatively, direct staining using fluorophore-conjugated markers, such as *Griffonia Simplicifolia* isolectin B4 and wheat germ agglutinin, can label blood vessels and cardiac sarcolemma, respectively. These markers usually have limited specificity, and their efficacy varies across tissues and species^12–14^. In contrast, single immunostaining of basement membrane protein provides an easy and robust way to label CMs and capillaries simultaneously^10,15,16^. However, the requirement of knowledge in identifying CMs and capillaries discourages the use of single-color immunostaining in quantitative image analysis. As deep-learning-assisted image analysis becomes widely available, the above-mentioned hurdle can potentially be overcome. Automatic classification and segmentation of CMs and capillaries on single-color immunofluorescence images of basement-membrane-stained sections will improve the throughput of quantitative capillarization assessment in the myocardium. This, in turn, will expedite cardiovascular research.

Deep learning (DL) approaches have been implemented to automate the classification and segmentation of biological structures in microscopic images^17–19^, majorly involving semantic and instance segmentation. Instance segmentation goes beyond semantic segmentation, since its capability to identify each object allows for counting objects under specific labels. Mask R-CNN^20^, PANet^21^, and YOLO-series (after YOLOv5)^22^ are among the most popular instance segmentation models. These models rely on end-to-end learning from large-scale datasets, requiring laborious data acquisition and pixel-wise annotation to achieve sufficient training. Like many other applications, DL in microscopy image analysis faces challenges due to limited training datasets. Inspired by prompt-based learning in natural language processing^23^, adapting large pre-trained vision models to downstream tasks via prompt engineering has emerged as a prominent approach to achieve few- or zero-shot transfer, i.e., adapting models to new scenarios with few or no labeled data^24^. Recently, a pre-trained image segmentation model, Segment Anything Model (SAM)^25^, has been demonstrated with impressive zero-shot transfer capability to new datasets via prompt engineering. However, SAM lacks classification capability; additional networks need to be developed to complete the task of classification and segmentation of CMs and capillaries.

**Figure 1:**
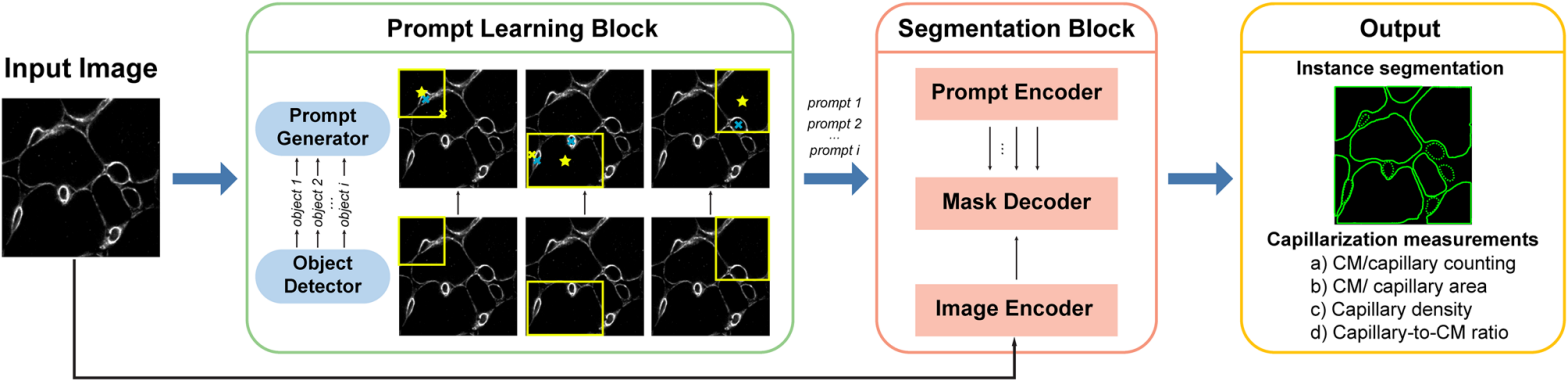
Overview of AutoQC and its sub-blocks. An immunofluorescence image of basement membrane is fed into both the prompt-learning block and the SAM-based segmentation block. The prompt-learning block is responsible for detecting objects, generating prompts, and classifying objects. The segmentation block is prompted for zero-shot transfer to new dataset. Commonly used capillarization-related measurements can be derived from segmentation results. Adapting SAM through prompt engineering converts a segmentation task into an object detection task, enabling weakly supervised learning.

Here, we propose a DL approach, named AutoQC, for automatic identification and segmentation of CMs and capillaries in immunofluorescence images of basement membrane in myocardial slices. AutoQC features a weakly supervised instance segmentation algorithm by integrating a SAM-based segmentation block with a custom-designed prompt-learning block (Fig. 1). The introduction of the prompt-learning block effectively adapts SAM for microscopic bioimages and enables AutoQC to classify objects. Moreover, common capillarization-related measurements can be computed and output by AutoQC. An ablation study was conducted to evaluate the effectiveness of the prompt design in AutoQC. The performance of AutoQC in instance segmentation and capillarization assessment was compared with SAM without prompt-learning (SAM-Only) and a state-of-the-art instance segmentation model, YOLOv8-Seg. The high throughput and accuracy achieved by AutoQC suggest that automatic quantification of cardiac capillarization can be performed efficiently with less staining and analysis effort.

## Results

### Ablation study

We conducted an ablation study to demonstrate the effectiveness of our prompt-design approach. The prompt-learning block of AutoQC consists of a YOLOv8-based object detector^22^ and a prompt generator. The object detector is responsible for identifying, locating, and classifying objects, as well as predicting bounding boxes. To ensure SAM can be properly prompted when segmenting densely organized structures in microscopic bioimages, the prompt generator was designed to output bounding boxes along with sets of binary-labeled points, serving as fine constraints (see “Design of the prompt-learning block”). The SAM model prompted by sets of binary-labeled points was named P-SAM, while the SAM model prompted by bounding boxes was named BB-SAM. P-SAM and BB-SAM shared the same object detector as AutoQC. The object detector was trained by bounding-box-annotated immunofluorescence images (see “Image acquisition and data annotation”). The original training dataset (*N*_*ori*_ = 67) was augmented and expanded to a total of 209 samples (*N*_*aug*_ = 209) for training (see “Model setup”). Five test runs were conducted to compare the instance segmentation performance among P-SAM, BB-SAM, and AutoQC (see “Statistics”).

**Figure 2:**
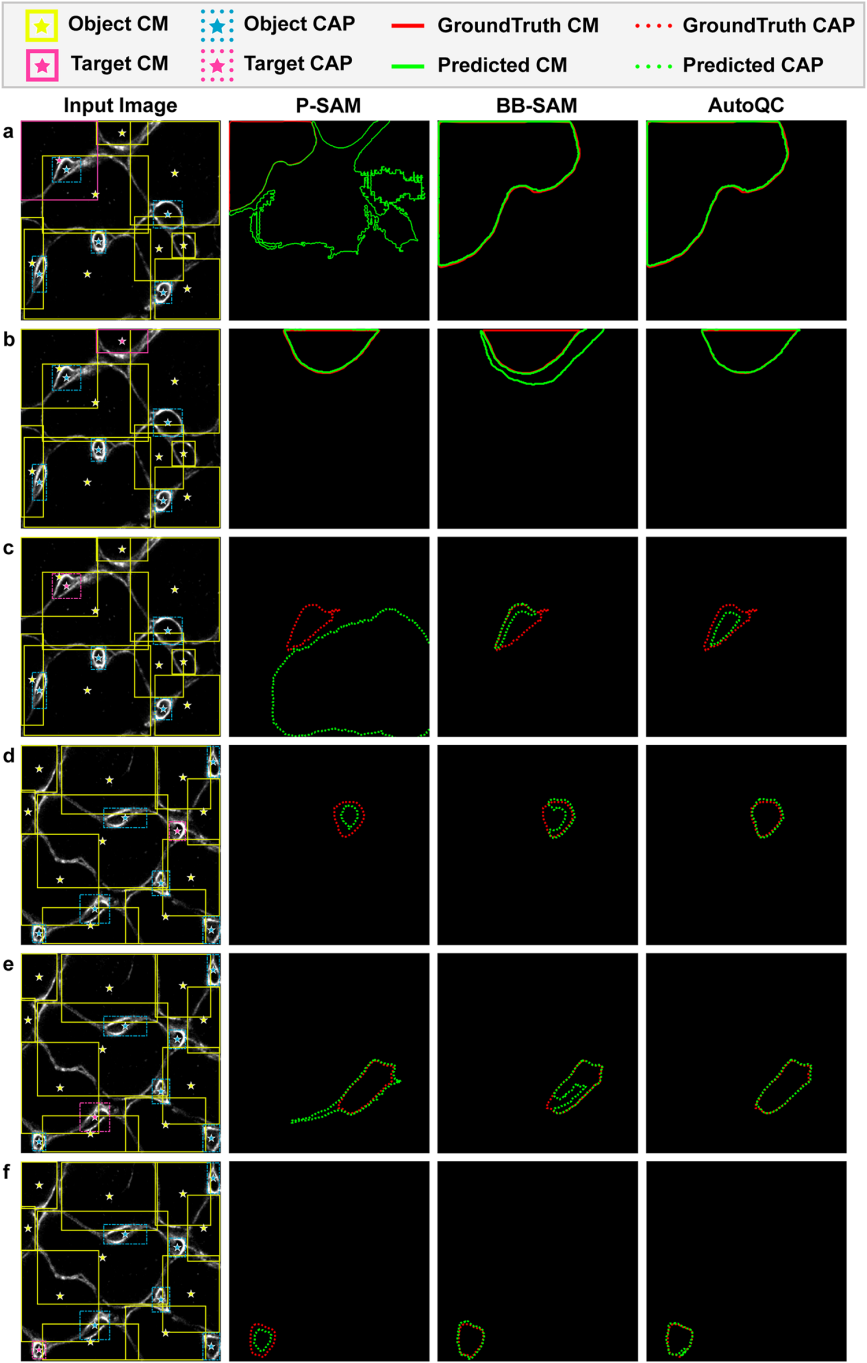
Representative segmentation results predicted by P-SAM, BB-SAM, and AutoQC in the ablation study. Segmentation results of two images ((**a**)–(**c**) for one and (**d**)–(**f**) for the other) from different test runs are presented. For each input image, the predicted masks (green) of three target objects, either CM or capillary, are displayed and overlaid with the ground truth masks(red). The bounding box and its centroid of each target object are highlighted (pink) in the input image. CM, objects with a cardiomyocyte label denoted by solid line; CAP, objects with a capillary label denoted by dashed line.

**Figure 3:**
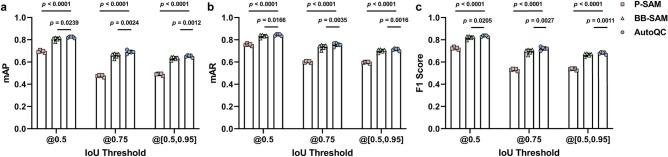
Quantitative evaluation of instance segmentation among P-SAM, BB-SAM, and AutoQC in the ablation study. (**a**) mAP, (**b**) mAR, and (**c**) F1 score were computed using IoU threshold 0.5, 0.75, and [0.5:0.95] with a step of 0.05. Paired two-tailed *t* tests were used to compare evaluation results between AutoQC and P-SAM or BB-SAM. *p* < 0.05 was considered significantly different.

Figure 2 shows representative segmentation masks predicted by P-SAM, BB-SAM, and AutoQC. P-SAM demonstrated limited capability of handling densely packed objects, while BB-SAM had improved performance in object localization and boundary detection. With enhanced prompts, the combination of binary-labeled keypoints and bounding boxes, AutoQC demonstrated better segmentation performance than P-SAM and BB-SAM. Since all models shared the same object detector, there was no performance difference in object classification (CM or capillary). For each test run, mAP (Fig. 3a), mAR (Fig. 3b), and F1 score (Fig. 3c) were computed on the test dataset (*N*_*test*_ = 32). BB-SAM outperformed P-SAM in terms of mAP, mAR, and F1 score. AutoQC achieved significantly higher mAP, mAR, and F1 score than P-SAM (all *p* values < 0.0001) and BB-SAM (all *p* values < 0.05). Moreover, AutoQC demonstrated the highest mAP, mAR, and F1 score (0.824, 0.844, and 0.834) when computed using the IoU threshold of 0.5. Evaluation results are reported as mean ± standard deviation (SD) in Supplementary Table 1.

### Benchmarking

AutoQC was benchmarked against YOLOv8-Seg, a state-of-the-art end-to-end model for instance segmentation. YOLOv8-Seg shared the same augmented dataset as AutoQC (*N*_*aug*_ = 209). In contrast to AutoQC, which used bounding-box-annotated images for its object detector training, YOLOv8-Seg used pixel-wise-annotated images during training (Supplementary Fig. 1). Moreover, YOLOv8-Seg shared the same setup as the YOLOv8-based object detector in AutoQC (see “Model setup”). We also compared AutoQC with SAM-Only, i.e., SAM without prompt-feeding. It should be noted that we randomly assigned a label to each mask predicted by SAM-Only as SAM is incapable of classifying objects. Five test runs were conducted to ensure fairness and reliability when comparing different models.

**Figure 4:**
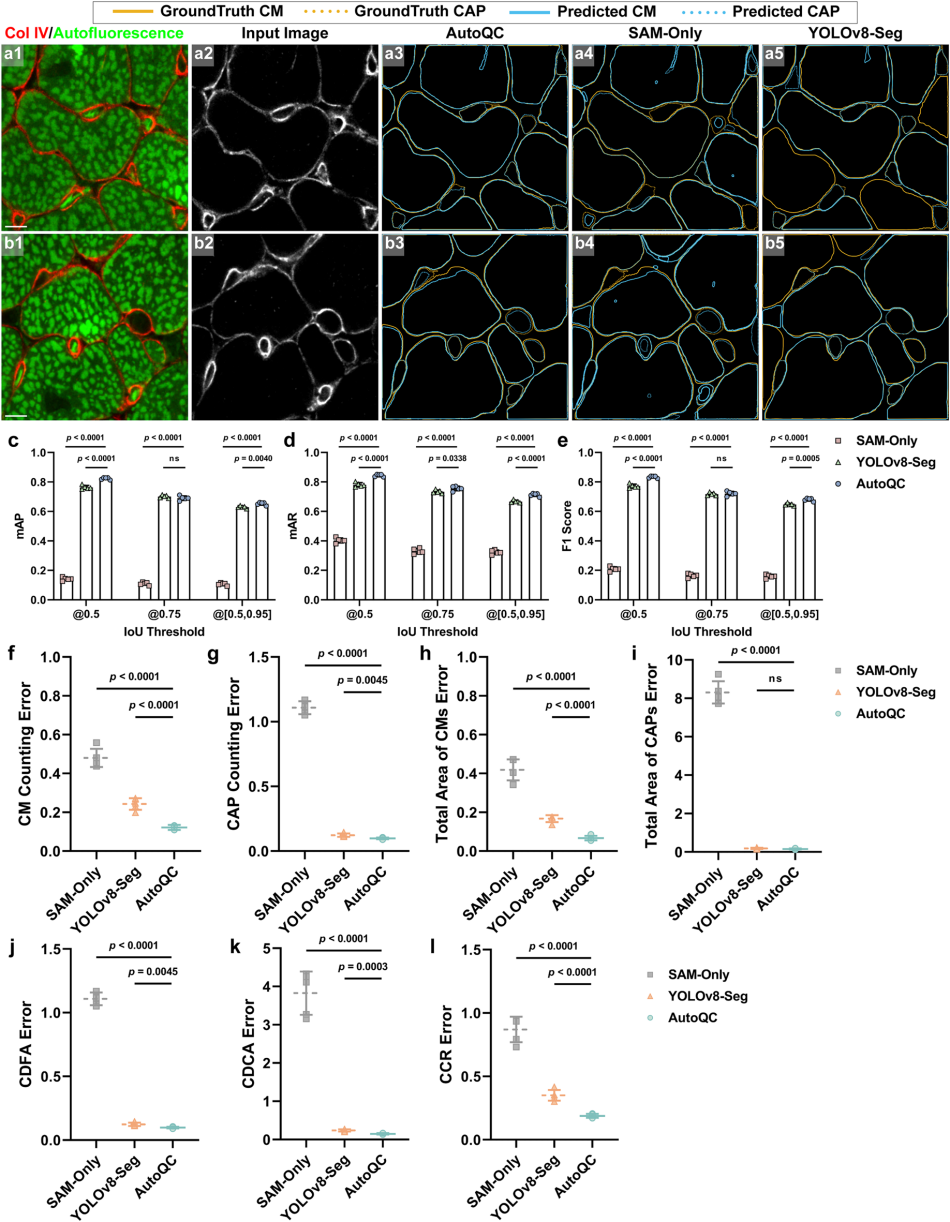
Performance evaluation among AutoQC, SAM-Only, andYOLOv8-Seg. (**a**, **b**) Representative segmentation results of two images from different test runs. (**a1**, **b1**) Immunofluorescence images of collagen type IV (ColIV, red) are merged with autofluorescence images (green) to facilitate the interpretation of CMs and capillaries in (**a2**, **b2**) input images. Predicted masks output by (**a3**, **b3**) AutoQC, (**a4**, **b4**) SAM-Only, and (**a5**, **b5**) YOLOv8-Seg are overlaid with the ground truth. CM, objects with a cardiomyocyte label denoted by solid line; CAP, objects with a capillary label denoted by dashed line; Scale bars, 5 μm. (**c**) mAP, (**d**) mAR, and (**e**) F1 score were computed using IoU threshold 0.5, 0.75, and [0.5:0.95] with a step of 0.05. The relative errors of the assessment on (**f**) CM counting, (**g**) capillary counting, (**h**) the total area of CMs, (**i**) the total area of capillaries, (**j**) CDFA, (**k**) CDCA, and (**l**) CCR. Error bars represent the standard deviation of the relative error (*n* = 5); unpaired two-tailed *t* tests; *p* < 0.05 was considered significantly different.

Figure 4a and b shows representative segmentation results predicted by AutoQC (Fig. 4a3,b3), SAM-Only (Fig. 4a4,b4), and YOLOv8-Seg (Fig. 4a5,b5). To facilitate interpreting CMs and capillaries in immunofluorescence images of basement membrane (Fig. 4a2,b2), autofluorescent features (Fig. 4a1,b1) as fiducial features are provided^26^. SAM-Only demonstrated inaccurate segmentation, especially for small objects, and ineffective classification. YOLOv8-Seg showed better performance in segmentation and classification; however, it failed to detect all objects within input images. AutoQC demonstrated the best overall segmentation performance, although CMs and capillaries without clear edges presented a challenge. We evaluated segmentation performance using metrics mAP (Fig. 4c), mAR (Fig. 4d) and F1 score (Fig. 4e). AutoQC demonstrated significantly higher mAP, mAR, and F1 score than SAM-Only (all *p* values < 0.0001) and YOLOv8-Seg (most *p* values < 0.005). AutoQC achieved the highest mAP, mAR, and F1 score, when calculated using IoU threshold of 0.5 (0.824, 0.844, 0.834) and IoU threshold range [0.5:0.95] (0.653, 0.713, 0.680). For the specific case, IoU threshold of 0.75, mAP and F1 score of YOLOv8-Seg were not significantly different from those of AutoQC; nevertheless, YOLOv8-Seg had a significantly lower mAR (*p* = 0.0338). The evaluation results of instance segmentation among three models are provided in Supplementary Table 1.

### Evaluation of AutoQC’s performance in capillarization assessment

We further evaluated the performance of capillarization assessment using various capillarization-related measurements derived from segmentation results, including the total number of CMs, the total number of capillaries, the total area of CMs (μm^2^), the total area of capillaries (μm^2^), capillary density normalized to field-of-view (CDFA, μm^−2^), capillary density normalized to CM area (CDCA, μm^-2^), and capillary-to-CM ratio (CCR) (see “Performance evaluation of capillarization assessment”). The relative errors of assessment were calculated by comparing these model-predicted measurements to the ground truth derived from manual segmentation masks and normalizing them to the ground truth (Fig. 4f–l). SAM-Only had the highest counting errors (CMs, 0.480 ± 0.0468; capillaries, 1.110 ± 0.0498). AutoQC demonstrated the lowest counting errors (CMs, 0.122 ± 0.0131; capillaries, 0.098 ± 0.0062), which were significantly lower than those of YOLOv8-Seg (CMs, 0.243 ± 0.0298; capillaries, 0.124 ± 0.0131). Moreover, AutoQC outperformed in measuring the total area of CMs, with an error of 0.067 ± 0.0115; the total area of capillaries, with an error of 0.157 ± 0.0299. We found that YOLOv8-Seg had no significantly different performance in measuring the total area of capillaries compared to AutoQC, with an error of 0.185 ± 0.0333. When assessing capillary density, AutoQC demonstrated the lowest errors (CDFA, 0.098 ± 0.0062; CDCA, 0.148 ± 0.0186). YOLOv8-Seg showed significantly higher errors when computing CDFA (0.124 ± 0.0131) and CDCA (0.240 ± 0.0288). SAM-Only had dramatically higher CDFA and CDCA errors with values of 1.110 ± 0.0498 and 3.830 ± 0.5650, respectively. Similarly, when quantifying CCR, AutoQC achieved an error of 0.189 ± 0.0154, significantly lower than that of YOLOv8-Seg (0.351 ± 0.0423) and SAM-Only (0.870 ± 0.1010). The relative errors in each test run are reported in Supplementary Table 2. Compared to the measurements derived from manually segmented masks, AutoQC achieved an average error below 0.125 across all categories of capillarization-related measurements. The average error for YOLO-v8-Seg reached 0.205, while SAM-Only, which lacks object classification capability, had a significantly higher average error of 2.304.

## Discussion

We proposed an automatic image analysis method, AutoQC, to quantitatively assess cardiac capillarization in basement-membrane-immunostained myocardial samples. We have demonstrated that AutoQC can effectively identify, classify, and segment CMs and capillaries in immunofluorescence images of basement membrane protein, collagen type IV.

To zero-shot transfer SAM to our task, i.e., segmenting CMs and capillaries in immunofluorescence images, we employed prompt engineering by enhancing bounding boxes with sets of binary-labeled centroids, serving as fine constraints. In the ablation study, we demonstrated this prompt design can effectively adapt SAM to microscopic bioimages and solve segmentation problems without additional training of SAM. Prompting SAM with bounding boxes alone was found to yield better performance compared to using sets of binary-labeled centroids alone. This can be attributed to the fact that centroids, as the internal keypoints of objects, lack the information of object boundaries, although they define the relative location of target and adjacent objects. Keypoint-based detectors, such as detecting each object as a triplet with one center point and two corners^27^ or five keypoints including extreme points and one center point^28^, have reported to be effective in object detection. Hence, it is possible to improve P-SAM performance by optimizing the design of keypoints, involving the implementation of keypoint detectors. Further study is needed to assess the performance improvement that optimizing keypoints may bring to AutoQC.

Although trained on a small dataset, AutoQC demonstrated impressive instance segmentation performance on microscopic images. AutoQC achieved significantly higher mAP, mAR, and F1 score compared to the state-of-the-art model YOLOv8-Seg. Furthermore, AutoQC showed promising performance in computing capillarization-related measurements, yielding a CDFA error of 0.098, a CDCA error of 0.148, and a CCR error of 0.189. In contrast, SAM-Only showed limitations on handling densely packed objects, leading to inaccurate segmentation. This result highlights the importance of prompting for transferring SAM to new tasks, especially in challenging scenarios, such as images with densely packed objects. It should be noted that SAM alone is unable to classify objects, making additional models (YOLOv8-based object detector in this work) essential for classification purposes. Segmenting small objects (capillaries) is more challenging compared to segmenting large objects (CMs). Expanding training dataset should help improve AutoQC’s instance segmentation performance on capillaries. Although it indeed involves increased work of image acquisition and data annotation, the weak annotation process reduces the overall workload compared to the pixel-wise annotation required by end-to-end segmentation models.

We demonstrated AutoQC can effectively identify and segment CMs and capillaries in immunofluorescence images of collagen type IV, a predominant basement membrane component in the myocardium. Alternatively, laminins, another major component of basement membrane, can be labeled for detection of CMs and capillaries. It should be noted that only laminin isoforms expressed in basement membrane surrounding CMs and underlying endothelial cells should be considered, such as isoforms containing subunit α4 and/or γ1^29–31^. AutoQC holds the potential for analyzing immunofluorescence images of these structural proteins, as long as the protein distribution encloses both CMs and capillaries. In addition to current application in assessing cardiac capillarization, AutoQC potentially can perform quantitative evaluation of capillarization/vascularization in other tissues, which requires further validation and algorithm optimization.

Confocal fluorescence microscopy allows 3D visualization of proteins of interest due to its optical-sectioning capability. By collecting Z stacks, AutoQC has the potential to analyze each image within the stack, allowing for 3D capillarization assessment. Image quality is an important factor which can affect the segmentation performance of models. To comprehensively evaluate AutoQC’s generalizability, it is essential to validate its performance on cross-modality data, such as widefield fluorescence images, and even brightfield images of immunoenzyme staining. Moreover, using a low-magnification objective lens provides larger FOV and captures more information than a high-magnification objective, although at the cost of image resolution. Validating AutoQC’s performance on low-resolution, large-FOV images will benefit studies with a large sample size.

In summary, we have developed AutoQC, an image analysis tool for automatic capillarization assessment of basement-membrane-immunostained myocardial samples. CMs and capillaries were labeled by immunofluorescence staining of basement membrane protein, collagen type IV in this work. Immunostained myocardial slices were imaged using confocal fluorescence microscopy. AutoQC automatically classified and segmented each CM and capillary within input immunofluorescence images. Capillarization-related measurements were computed from segmented masks, including the total number and occupied area of each category (CM and capillary), as well as capillary density (CDFA, CDCA, and CCR). We have demonstrated that AutoQC outperformed the state-of-the-art instance segmentation model, YOLOv8-Seg. Moreover, AutoQC was trained on a small dataset with bounding box annotations, reducing the workload associated with data acquisition, manual annotation, and model training. As such, AutoQC provides an efficient and practical solution for quantitative assessment of cardiac capillarization when performing immunostaining of basement membrane on myocardial samples. This tool will make single immunostaining of basement membrane a high-throughput and cost-effective solution for evaluating cardiac capillarization, especially in studies when double immunostaining is challenging and/or in studies with a large sample size.

## Methods

### Myocardial slice preparation

All procedures on mice and animal care were approved by the Institutional Animal Care and Use Committee (IACUC) of the Medical University of South Carolina (protocol number, IACUC-2019-00868). All animal experiments were performed in accordance with ARRIVE guidelines and local regulations. C57BL/6 mice were placed in a euthanasia chamber, and euthanasia was conducted by gradually filling the chamber with 100% CO_2_ for at least 3 min. Ventricles were isolated from the hearts of three 5-month female littermates, fixed in freshly prepared 4% (w/v) paraformaldehyde, cryoprotected by sucrose solution, then snap-frozen in liquid-nitrogen-cooled isopentane. Frozen blocks were consecutively sectioned into 10 µm-thick slices and mounted on microscope slides (VWR International, Radnor, PA). Myocardial slices obtained from each mouse ventricle were randomly selected for subsequent immunostaining.

### Immunofluorescence

Heat-induced antigen retrieval was performed in sodium citrate pH 6.0 at 95 °C. Slices were blocked in 2% bovine serum albumin with 0.3% Triton X-100 at room temperature, then incubated with antibodies to collagen type IV (ab19808; Abcam, Boston, USA) at 4 °C. Slices were stained with abberior STAR RED (STRED; abberior, Göttingen, Germany) at room temperature. After staining, slices were mounted in custom-made Mowiol medium and coverslipped. Primary and secondary antibodies were diluted in the blocking buffer. Negative-primary-antibody controls were performed to confirm the staining signal was from the detection of target antigens.

### Home-built confocal fluorescence microscope

Confocal fluorescence imaging was performed on a home-built multimodal microscope^32^. Briefly, two picosecond pulsed lasers (470 nm and 635 nm; PicoQuant) were synchronized, serving as excitation. Two excitation beams were combined by dichroic mirrors (FF662-FDi01 and Di02-R488; Semrock), deflected by an XY two-axis galvanometer set (8310 K; Cambridge Technology), relayed by a lens pair (89683, Edmund Optics; TTL200, Thorlabs), directed to the back aperture of an HCX Plan Apo 63 × oil NA 1.40 objective (Leica Microsystems).

For the detection of Abberior-STAR-RED-stained collagen type IV, the excitation laser at 635 nm was used. The emission light was spectrally separated from the excitation lights and green fluorescence by dichroic mirrors (Di02-R488, FF662-FDi01 and FF735-Di01; Semrock), filtered by a bandpass filter (690/50; Chroma), coupled into a multimode fiber (M31L01; Thorlabs), and detected by a single photon detector (SPCM-AQRH-13; Excelitas Technologies Corp.). For the detection of green autofluorescence in formaldehyde-fixed myocardial slices, the excitation laser at 470 nm was used. The emission light was spectrally separated from the excitation lights and red fluorescence by dichroic mirrors (Di02-R488, FF662-FDi01 and FF624-Di01; Semrock), filtered by a bandpass filter (FF01-525/50; Semrock), coupled into a multimode fiber (M42L01; Thorlabs), and detected by a single photon detector (SPCM-AQRH-13; Excelitas Technologies Corp.).

### Image acquisition and data annotation

Immunofluorescence and autofluorescence images were sequentially acquired in a frame-scan mode using SciScan (Scientifica, Uckfield, UK). All samples were imaged under the same experimental conditions of excitation and detection. Each region of interest (ROI) was imaged with a constant field of view (FOV), 42.5 μm × 42.5 μm. ROIs were selected based on the criterion that at least 80% FOV contained myocardial structures, specifically CMs and capillaries at myocardial cross-sectioned area. A total of 107 ROIs were imaged (*N* = 107). Raw images (512 × 512 pixels, 16-bit grayscale) were pre-processed by Wiener filtering (kernel size, 3 × 3) and contrast enhancement using a custom MATLAB script. Immunofluorescence images were manually segmented by two observers, reaching a consensus. During this process, features in autofluorescence images were used as fiducial features to facilitate the identification of CMs and capillaries in immunofluorescence images of basement membrane^26^. Bounding box annotations for each image were generated by circumscribing manually segmented masks with rectangular boxes using a custom Python script. For the training of the object detector in AutoQC’s prompt-learning block, bounding-box-annotated immunofluorescence images with labels (CM or capillary) were utilized. For the training of the end-to-end instance segmentation model (YOLOv8-Seg), pixel-wise-annotated immunofluorescence images with labels (CM or capillary) were utilized. The training workflow for the weakly supervised algorithm AutoQC and the end-to-end model YOLOv8-Seg is summarized in Supplementary Fig. 1.

### AutoQC algorithm design

#### Overall architecture

AutoQC harnesses the power of SAM and enables zero-shot transfer to our segmentation task by an automated prompt-learning block with classification capability. The input image is a grayscale immunofluorescence image, where CMs and capillaries are outlined by immunostaining of collagen type IV (basement membrane protein). The input image is processed by the prompt-learning block to generate prompts and assign labels (CM or capillary) for each detected object. Together with the input image, prompts and their labels are fed into the SAM-based segmentation block. The segmentation block outputs predicted segmentation masks. Meanwhile, commonly used capillarization-related measurements can be computed from segmentation results. An algorithm diagram is provided in Supplementary Fig. 2.

#### Design of the prompt-learning block

The prompt-learning block is designed for complete automation without the need for manual prompt crafting. The prompt-learning block consists of an object detector and a prompt generator. The YOLOv8-based object detector^22^ is responsible for detecting and classifying objects. For each detected object, a bounding box with a label is output. An object detection task requires less annotated data for training compared to a segmentation task. Furthermore, bounding boxes are defined by corner points, allowing for lightweight storage and faster operation.

**Figure 5:**
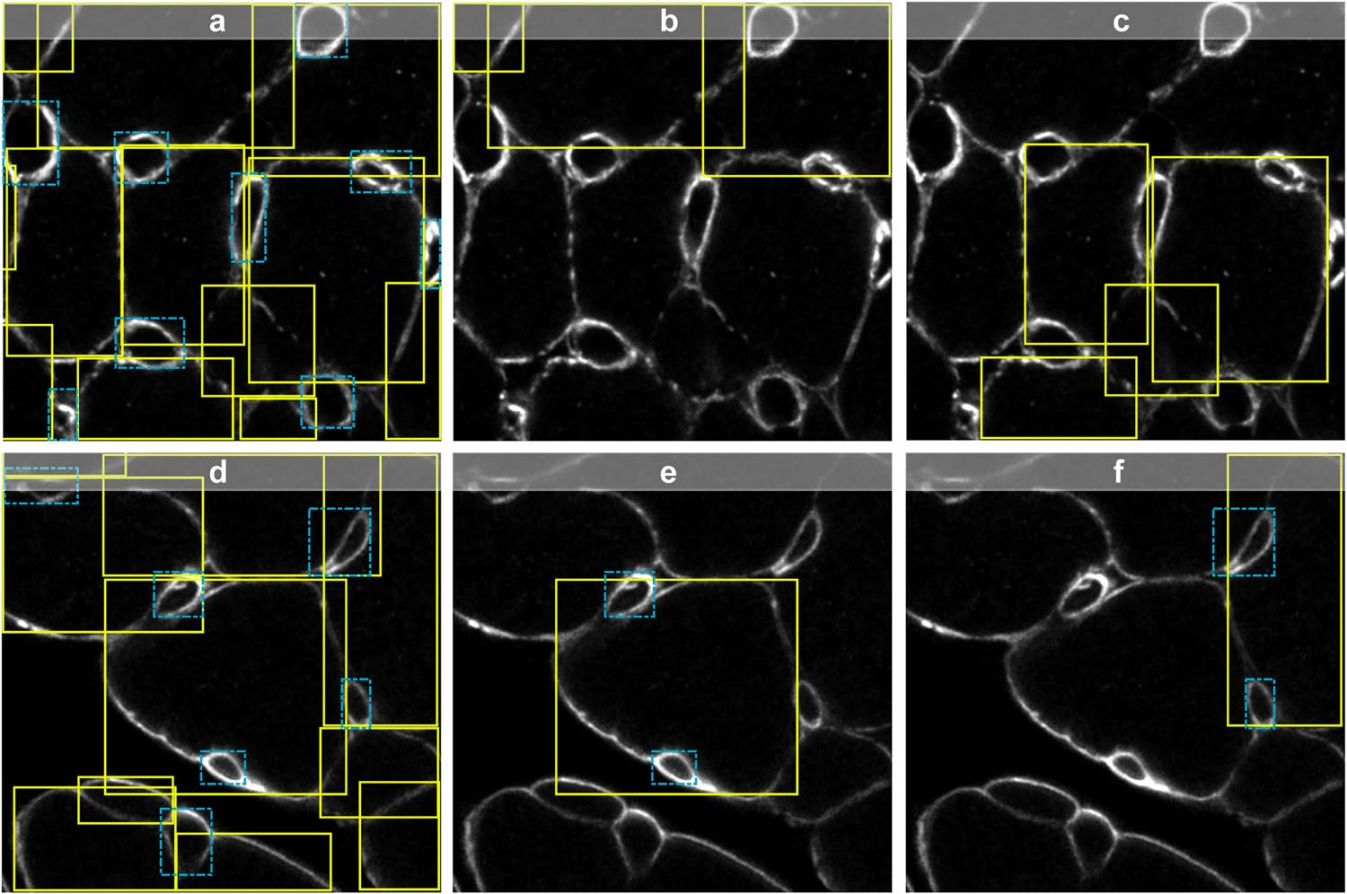
Representative immunofluorescence images of densely packed CMs and capillaries. Bounding boxes of CMs are marked by solid lines (yellow), and those of capillaries are marked by dashed lines (blue). (**a**, **d**) Densely packed CMs and capillaries result in largely overlapped bounding boxes. (**b**, **c**) Pronounced overlap among the bounding boxes of adjacent CMs. (**e**, **f**) Some bounding boxes of capillaries are fully enclosed within the bounding boxes of nearby CMs.

In scenarios where objects are sparsely distributed within images, the overlap of bounding boxes is negligible. Hence, directly employing bounding boxes as prompts can effectively zero- or few-shot transfer pre-trained models to custom datasets. However, biological structures typically organize in a dense manner and exhibit complicated protein patterns in microscopic bioimages. Within the myocardium, CMs are tightly packed, and each CM is surrounded by multiple capillaries. The predicted bounding boxes of CMs and capillaries demonstrated pronounced overlap (Fig. 5). Due to the non-convex hull shape of CMs, the bounding boxes of adjacent CMs always overlapped with each other (Fig. 5b,c). Moreover, the bounding boxes of capillaries were frequently enclosed by nearby CMs (Fig. 5e,f). We found that direct prompting SAM with these bounding boxes had an adverse effect on segmentation performance. As such, we designed a prompt generator to ensure prompts properly function when adapting SAM to the custom dataset.

For a detected object in the input image, the bounding box ${{\varvec{B}}}_{i}$ works as coarse constraint that can limit the maximum range of the segmentation mask and provide the segment-based label. Each bounding box ${{\varvec{B}}}_{i}$ has a centroid, ${c}_{i}$. To ensure a bounding box properly functions when prompting SAM, a set of binary-labeled points ${{\varvec{P}}}_{i}$ is generated as fine constraint. The point set ${{\varvec{P}}}_{i}$ is generated by traversing centroids of all detected objects in the image and determining if they fall within the region of ${{\varvec{B}}}_{i}$. Centroids located within the region of ${{\varvec{B}}}_{i}$ are declared as the elements of ${{\varvec{P}}}_{i}$, with assigned binary labels. For all possible elements ${c}_{j}$ in the point set ${{\varvec{P}}}_{i}$, if $j \ne i$, ${c}_{j}$ is declared as $\{{c}_{j}, 0\}$; if $j = i$, ${c}_{j}$ is declared as $\{{c}_{j}, 1\}$. Supplementary Fig. 3 shows engineered prompts for detected objects in an example image. This prompt-design approach requires a maximal complexity of $O({n}^{2})$ for segmenting an image, where *n* refers to the number of detected objects in the image.

#### SAM-based segmentation block

The SAM-based segmentation block consists of an image encoder, a prompt encoder, and a mask decoder. The image encoder for generating embeddings of the input image is based on a masked autoencoder^33^. The prompt encoder for creating embeddings of the prompts generated by the prompt-learning block is based on CLIP^34^. The mask decoder is based on a conventional transformer decoder^35^, utilizing both image and prompt embeddings to predict segmentation masks. It should be noted that each prompt corresponds to a mask prediction. In this study, we utilized the pre-trained model weights of SAM and did not perform any tuning.

### Model setup

All training, validation, and testing were executed on one node of the Palmetto cluster (Clemson University, Clemson, USA) with an Intel® Xeon® Platinum 8358 processor and an NVIDIA® A100 GPU. Two models, the YOLOv8-based object detector in AutoQC and YOLOv8-Seg, were trained using the stochastic gradient descent optimizer with a learning rate of 0.01. A momentum of 0.937 was incorporated into the training process, allowing for faster convergence and reduced oscillation. The mini-batch size was set to 16 and each model was trained for 100 epochs. The image dataset (*N* = 107) was split into 70% for training/validation and 30% for testing (*N*_*test*_ = 32). 10% of the training/validation dataset was used for validation (*N*_*val*_ = 8). The original training dataset (*N*_*ori*_ = 67) after random rotation and mosaic augmentation was expanded to a total of 209 samples (*N*_*aug*_ = 209). Random rotation was utilized in three directions: 90-degree clockwise, 90-degree counter-clockwise, and 90-degree upside-down. Mosaic augmentation combined four images into a single image, by placing each image into a quadrant of the new image. The model weights of each model were optimized on the validation dataset.

### AutoQC performance analysis

#### Performance evaluation of instance segmentation

COCO standard metrics^36^ were utilized to evaluate segmentation performance. Mean average recall (mAR), mean average precision (mAP), and F1 score were computed across all labels. Intersection over Union (IoU) is commonly used to measure the degree of overlap between a predicted mask (*M*_*p*_) and the ground truth (*M*_*gt*_). When IoU (*M*_*p*_, *M*_*gt*_) ≥ *ϕ*, the predicted mask with the identical label as the ground truth is considered a true positive (TP), where *ϕ* refers to a custom-defined IoU threshold. When IoU (*M*_*p*_, *M*_*gt*_) < *ϕ*, the predicted mask with the identical label as the ground truth is considered a false positive (FP). If the label of the predicted mask does not match the ground truth, the predicted mask is considered a true negative (TN) when IoU (*M*_*p*_, *M*_*gt*_) ≥ *ϕ*; a false negative (FN) when IoU (*M*_*p*_, *M*_*gt*_) < *ϕ*. Specifically, we used two single-value IoU thresholds, 0.5 and 0.75; one IoU threshold range [0.5:0.95] with a step size of 0.05. When computing segmentation metrics under the range [0.5:0.95], results of each metric were averaged across all labels and ten IoU thresholds within the range. Evaluation metrics used in this study are summarized in Supplementary Table 3. It should be noted that accuracy was not considered in this study to avoid biased performance evaluation. For instance, given a scenario where 9 capillaries and a single CM exhibit in an image, if a model correctly predicts all capillaries but fails to predict the only CM, its accuracy of 90% is misleading and insufficient to reflect the performance in predicting CMs.

#### Performance evaluation of capillarization assessment

From each image, the area of each object mask was measured in μm^2^, and the number of objects in each category (CM or capillary) was counted. The following capillarization-related measurements were computed for each image:

- The total number of CMs, was counted as the number of masks with a CM label.
- The total number of capillaries, was counted as the number of masks with a capillary label.
- The total area of CMs (μm^2^), was calculated as the area occupied by masks with a CM label.
- The total area of capillaries (μm^2^), was calculated as the area occupied by masks with a capillary label.
- Capillary density normalized to FOV area (CDFA, μm^−2^), was defined as capillaries per FOV area and calculated as the total number of masks with a capillary label divided by the FOV.
- Capillary density normalized to CM area (CDCA, μm^−2^), was defined as capillaries per CM area and calculated as the total number of masks with a capillary label divided by the total area of masks with a CM label.
- Capillary-to-CM ratio (CCR), was calculated as the ratio of the number of masks with a capillary label to the number of masks with a CM label.

For each capillarization-related measurement, the relative error of an assessment $\delta$ was calculated as,

$$\delta =\frac{\left|predicted \,result-ground \, truth\right|}{ground \, truth}$$

where the predicted result refers to the capillarization-related measurement output by a model, while the ground truth refers to the measurement derived from manual segmentation masks (pixel-wise annotations done by two observers after reaching the consensus).

### Statistics

Five test runs were conducted to ensure fairness and reliability when comparing different models. For each test run, the training/validation dataset was randomly shuffled before preparing augmented training dataset and validation dataset (see “Model setup”). Performance evaluation was conducted on the test dataset (*N*_*test*_ = 32) for each test run, and evaluation results (*n* = 5) were reported as mean ± SD. Statistical differences were examined using unpaired or paired two-tailed *t* tests in Prism 9 (GraphPad Software). A significance level of *p* < 0.05 was considered statistically significant.

### Supplementary Information

Supplementary Information.

## Data Availability

Image dataset is available from the corresponding author upon request. A test dataset is available in our github repository https://github.com/NFILab/AutoQC-code.

## Code availability

All code for the models used in this work is available in our github repository https://github.com/NFILab/AutoQC-code.
