# Supplementary material for: Quantification of cardiac capillarization in basement-membrane-immunostained myocardial slices using Segment Anything Model
Source: Sci Rep. 2024 Jul 3;14:15344. doi: 10.1038/s41598-024-65567-3 (PMC11222533; doi:10.1038/s41598-024-65567-3)
Supplement: Supplementary file 1 — Supplementary Information. [file 41598_2024_65567_MOESM1_ESM.docx]

**Supplementary Table 1.**

Instance segmentation evaluation results are reported as mean ± SD (*n* = 5).

|  | **SAM-Only** | **P-SAM** | **BB-SAM** | **YOLOv8-Seg** | **AutoQC** |
| --- | --- | --- | --- | --- | --- |
| mAP @0.5 | 0.141±0.0111 | 0.695±0.0100 | 0.807±0.0165 | 0.764±0.0132 | **0.824±0.0070** |
| mAP @0.75 | 0.109±0.0101 | 0.475±0.0090 | 0.658±0.0211 | **0.701±0.0099** | 0.690±0.0148 |
| mAP @[0.5:0.95] | 0.106±0.0090 | 0.489±0.0081 | 0.634±0.0146 | 0.630±0.0082 | **0.653±0.0098** |
| mAR @0.5 | 0.403±0.0166 | 0.756±0.0096 | 0.834±0.0130 | 0.781±0.0145 | **0.844±0.0086** |
| mAR @0.75 | 0.331±0.0175 | 0.602±0.0080 | 0.734±0.0212 | 0.734±0.0117 | **0.756±0.0153** |
| mAR @[0.5:0.95] | 0.323±0.0147 | 0.596±0.0073 | 0.702±0.0139 | 0.667±0.0091 | **0.713±0.0107** |
| F1 @0.5 | 0.209±0.0144 | 0.724±0.0097 | 0.820±0.0148 | 0.772±0.0137 | **0.834±0.0078** |
| F1 @0.75 | 0.164±0.0135 | 0.531±0.0086 | 0.694±0.0212 | 0.717±0.0105 | **0.721±0.0148** |
| F1 @[0.5:0.95] | 0.159±0.0120 | 0.537±0.0078 | 0.665±0.0142 | 0.647±0.0083 | **0.680±0.0103** |

Note: The highest value in each evaluation metric category is bolded.

**Supplementary Table 2.**

Summary of assessment errors when predicting capillarization-related measurements (*n* = 5).

| **The total number of CMs** | | | | | | |
| --- | --- | --- | --- | --- | --- | --- |
|  | Test 1 | Test 2 | Test 3 | Test 4 | Test 5 | Mean ± SD |
| SAM-Only | 0.5590 | 0.4680 | 0.4378 | 0.4558 | 0.4816 | 0.480±0.0468 |
| YOLOv8-Seg | 0.2314 | 0.2667 | 0.1984 | 0.2719 | 0.2468 | 0.243±0.0298 |
| AutoQC | 0.1319 | 0.1294 | 0.1098 | 0.1065 | 0.1339 | **0.122±0.0131** |
| **The total number of capillaries** | | | | | | |
|  | Test 1 | Test 2 | Test 3 | Test 4 | Test 5 | Mean ± SD |
| SAM-Only | 1.0660 | 1.1418 | 1.1683 | 1.1156 | 1.0503 | 1.110±0.0498 |
| YOLOv8-Seg | 0.1219 | 0.1455 | 0.1122 | 0.1151 | 0.1235 | 0.124±0.0131 |
| AutoQC | 0.1067 | 0.0970 | 0.1002 | 0.0895 | 0.0983 | **0.098±0.0062** |
| **The total area of CMs** | | | | | | |
|  | Test 1 | Test 2 | Test 3 | Test 4 | Test 5 | Mean ± SD |
| SAM-Only | 0.3421 | 0.4042 | 0.4056 | 0.4718 | 0.4705 | 0.419±0.0542 |
| YOLOv8-Seg | 0.1762 | 0.1764 | 0.1360 | 0.1779 | 0.1729 | 0.168±0.0179 |
| AutoQC | 0.0649 | 0.0644 | 0.0547 | 0.0859 | 0.0645 | **0.067±0.0115** |
| **The total area of capillaries** | | | | | | |
|  | Test 1 | Test 2 | Test 3 | Test 4 | Test 5 | Mean ± SD |
| SAM-Only | 8.3749 | 9.2556 | 7.7251 | 8.1113 | 8.0698 | 8.310±0.5780 |
| YOLOv8-Seg | 0.1721 | 0.2005 | 0.1744 | 0.2325 | 0.1444 | 0.185±0.0333 |
| AutoQC | 0.1739 | 0.1604 | 0.1191 | 0.1367 | 0.1950 | **0.157±0.0299** |
| **Capillary density normalized to FOV area (CDFA)** | | | | | | |
|  | Test 1 | Test 2 | Test 3 | Test 4 | Test 5 | Mean ± SD |
| SAM-Only | 1.0660 | 1.1418 | 1.1683 | 1.1156 | 1.0503 | 1.110±0.0498 |
| YOLOv8-Seg | 0.1219 | 0.1455 | 0.1122 | 0.1151 | 0.1235 | 0.124±0.0131 |
| AutoQC | 0.1067 | 0.0970 | 0.1002 | 0.0895 | 0.0983 | **0.098±0.0062** |

(continued)

| **Capillary density normalized to CM area (CDCA)** | | | | | | |
| --- | --- | --- | --- | --- | --- | --- |
|  | Test 1 | Test 2 | Test 3 | Test 4 | Test 5 | Mean ± SD |
| SAM-Only | 3.1568 | 4.1097 | 4.3328 | 4.2575 | 3.2718 | 3.830±0.5650 |
| YOLOv8-Seg | 0.2633 | 0.2305 | 0.2018 | 0.2733 | 0.2291 | 0.240±0.0288 |
| AutoQC | 0.1322 | 0.1457 | 0.1287 | 0.1669 | 0.1679 | **0.148±0.0186** |
| **Capillary-to-CM ratio (CCR)** | | | | | | |
|  | Test 1 | Test 2 | Test 3 | Test 4 | Test 5 | Mean ± SD |
| SAM-Only | 0.9463 | 0.9439 | 0.9347 | 0.7930 | 0.7320 | 0.870±0.1010 |
| YOLOv8-Seg | 0.3517 | 0.3531 | 0.3018 | 0.4169 | 0.3314 | 0.351±0.0423 |
| AutoQC | 0.1896 | 0.2069 | 0.1751 | 0.1712 | 0.1998 | **0.189±0.0154** |

1) Assessment errors were calculated by comparing model-predicted measurements to the ground truth obtained from manual segmentation results, as $\left| predicted result - ground truth \right|/ground truth$.

2) The lowest error, mean ± SD, for each capillarization assessment is bolded.

**Supplementary Table 3.**

Segmentation evaluation metrics used in the study. (*N* refers to the number of images)

| **Metrics** | **Equations** |
| --- | --- |
| Intersection over Union (IoU) | $IoU=\frac{M_{p}\cap M_{gt}}{M_{p}\cup M_{gt}}$ |
| Mean Average Recall (mAR) | $mAR=1/N\sum_{i=1}^{N} {AR}_{i}$ |
| Mean Average Precision (mAP) | $mAP=1/N\sum_{i=1}^{N} {AP}_{i}$ |
| F1 Score | $F1 Score=2\times\frac{mAR\times mAP}{mAR+mAP}$ |

**Supplementary Figure 1.**


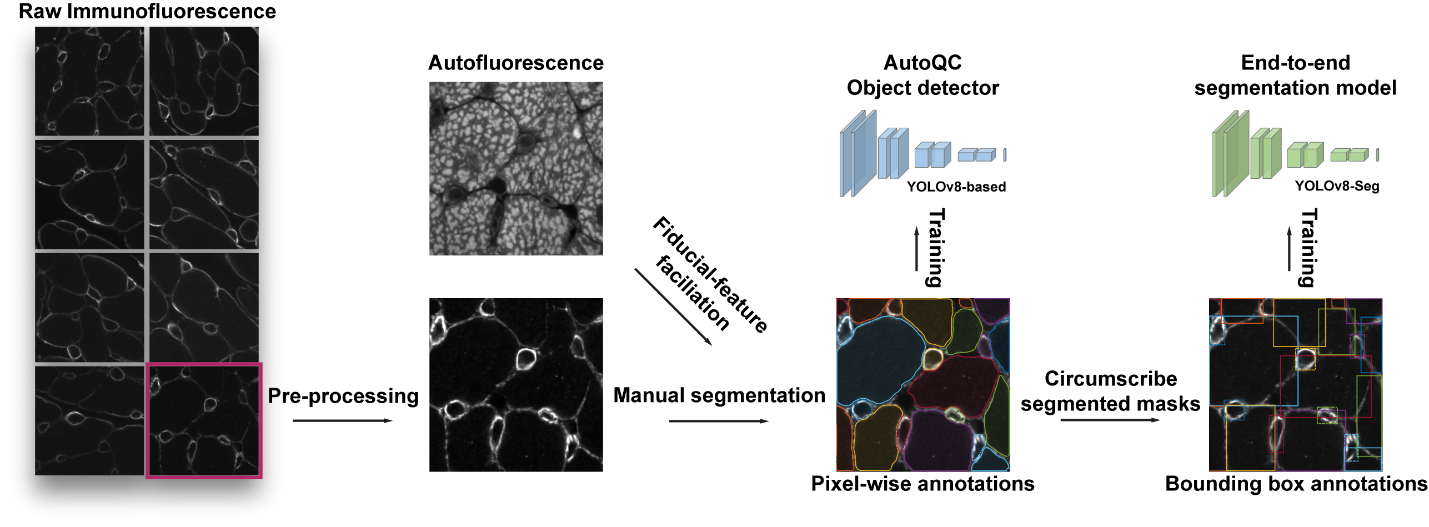


Training workflow for AutoQC’s YOLOv8-based object detector and the end-to-end instance segmentation model (YOLOv8-Seg). (1) Immunofluorescence images were pre-processed before manual segmentation. (2) With the aid of autofluorescent features, CMs and capillaries in basement-membrane-immunostained images were identified and segmented by two observers after reaching a consensus. (3) Immunofluorescence images were both bounding-box-annotated and pixel-wise-annotated. (4) Immunofluorescence images with weak annotations (bounding boxes) were used for training AutoQC’s object detector. The same image dataset with pixel-wise annotations was used for training YOLOv8-Seg.

**Supplementary Figure 2.**

**Supplementary Figure 3.**


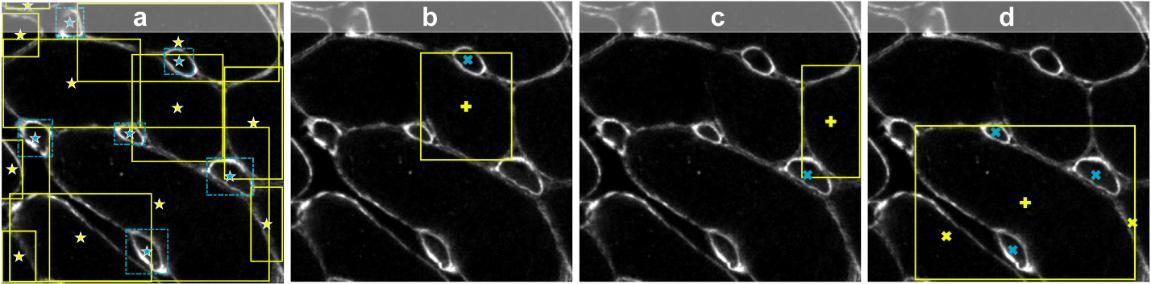


Example of engineered prompts for detected objects in a basement-membrane-stained immunofluorescence image. **(a)** Bounding boxes of objects with a CM label are drawn with solid lines (yellow), while those with a capillary label are drawn with dashed lines (blue). The centroids of bounding boxes are indicated by a pentagram sign with a matching color. **(b-d)** The prompt for a target object consists of a bounding box $\boldsymbol{B}_{i}$ and a set of binary-labeled points $\boldsymbol{P}_{i}$. The set of binary-labeled points $\boldsymbol{P}_{i}$ includes: the centroid of the target bounding box (*j* = *i*) declared as {$c_{i}$, **1**} (plus sign); the centroids of other bounding boxes (*j* ≠ *i*) that are located within the region of the target bounding box declared as {$c_{i}$, **0**} (cross sign).
